# Bacterial Infections: Surveillance, Prevention and Control

**DOI:** 10.3390/pathogens13020181

**Published:** 2024-02-17

**Authors:** Anna Maria Spagnolo

**Affiliations:** Department of Health Sciences, University of Genova, Via Pastore 1, 16132 Genova, Italy; am.spagnolo@unige.it

## 1. Background

Bacteria play a vital role in maintaining human health, but they may also be responsible for many different serious infections and diseases. Severe bacterial infections are characterized as having high morbidity and mortality rates; therefore, the appropriateness of therapy can have a profound clinical impact.

The severity of these infections is due to the virulence factors of the bacteria, their occurrence in debilitated patients, and their resistance to antibiotics, which involves the associated difficulty in treating infections.

Antibiotic resistance represents a severe problem in public health due to the increasing diffusion in the general population, animals and the environment of antibiotic-resistant bacteria (ARB) with resistance phenotypes extended to more than one class of antibiotics.

Owing to the widespread emergence of ARB (e.g., methicillin-resistant *Staphylococcus aureus*, vancomycin-resistant enterococci, carbapenemase-producing Enterobacterales, carbapenem-resistant Acinetobacter baumannii, multidrug-resistant *Pseudomonas aeruginosa*), the therapeutic benefits of antibiotics have been reduced [1,2,3,4].

*Pseudomonas aeruginosa* is one of the most frequent and severe causes of hospital-acquired infections, particularly affecting immunocompromised (especially neutropenic) and intensive care unit (ICU) patients. The majority of *P. aeruginosa* strains are resistant to most antibiotics currently in use [5,6,7].

Bacteria exploit various efficient strategies to neutralize the action of antibiotics, often leaving no effective treatment options to treat the infectious diseases caused by them. The continuous selective pressure exerted by antibiotics has helped bacteria develop resistance to one or more drugs simultaneously [8].

In Italy, antimicrobial consumption is among the highest in Europe and, according to ECDC, Italy continues to have the highest rate of antibiotic resistance for several pathogens.

Multidrug-resistant bacteria (MDROs) are continuing to develop and spread in the healthcare system, and so healthcare-associated infections (HAIs) pose one of the most severe threats to patients’ health and remain a major challenge for healthcare providers globally.

HAIs are infections acquired by patients during their stay in a hospital or another healthcare setting that were not present or incubating at the time of admission.

In the European Union and European Economic Area (EU/EEA), more than 3.5 million cases of HAIs (5.7–7.1% of all hospitalized patients) are estimated to occur each year, leading to more than 90 thousand deaths [9,10].

The increasing emergence in the hospital environment of MDROs, including resistance to last-resort drugs, such as carbapenems, is of particular concern and is increasingly being reported worldwide [11].

A worrying increase in bacterial resistance has been observed in recent years, particularly among Gram-negative bacteria (especially Enterobacterales). The phenomenon of multi-resistance has increased significantly in this group of microorganisms due to the spread of extended-spectrum betalactamases (ESBLs) and, more recently, carbapenemases. The latter are particularly dangerous because they make it difficult to treat infections caused by the microorganisms that produce them (e.g., *Klebsiella pneumoniae*) [12].

In 2015, WHO Member States unanimously endorsed a Global Plan of Action to address antimicrobial resistance (AMR), and WHO called on all countries to take concerted action across all sectors of human activity, with a One Health approach bringing together stakeholders from relevant sectors to communicate and work together in the design, implementation, and monitoring of programs, policies, legislation, and research to mitigate AMR and achieve better health and economic outcomes [13].

Adopting a surveillance system to monitor infection rates is the first step in identifying local problems and priorities and evaluating the effectiveness of infection control activities.

To estimate the overall impact of ICAs and antibiotic use in Europe, the ECDC proposed a European point prevalence study based on a standardized methodology that would allow comparison of the data collected from different countries.

The objectives of the European prevalence study of HAIs and of antibiotic use in acute care hospitals are as follows [14]:

1. To estimate the overall size (prevalence) of ICAs and antibiotic use in acute care hospitals in Europe.
2. Catalogue patients, invasive procedures, infections (sites and microorganisms involved, including antibiotic resistance markers) and antibiotics prescribed (molecules and indications for use) by the type of patient, ward, inpatient facility, and country through adjusted or stratified data.
3. Disseminate the results to those the appropriate parties, i.e., locally, regionally, national and European levels; facilitate greater attention towards the problem; promote and strengthen infrastructure and expertise to implement surveillance; identify common problems at the European level and establish shared priorities; evaluate the impacts of the strategies and target policies at the local/national/regional level (point prevalence study must be repeated in all member countries).
4. Provide a standardized tool for hospitals to identify targets for quality improvement.

The latest version of the ECDC protocol (version 6.1, ECDC PPS 2022–2023) includes changes to the third ECDC point prevalence study organized in 2022 and 2023. The main changes are the inclusion of healthcare-associated COVID-19 and related indicators, the simplification of the antimicrobial use data, the inclusion of indicators on automated HAI surveillance, and an alignment of the question regarding multimodal strategies for the implementation of infection prevention and control interventions [9].

In addition to epidemiological surveillance in healthcare setting, it is also important to surveil the microbial activity in the environment, water and employed devices (e.g., duodenoscopes) [15,16,17,18,19,20,21,22].

To control and reduce healthcare infections, action is needed on several fronts, including the following: (i) the identification of dedicated staff, such as an Infection Control Committee and surveillance nurses; (ii) proper use of antibiotics; (iii) establishment of active infection surveillance protocols and appropriate information flows to identify infections; (iv) proper training of dedicated staff to treat patients, especially in the critical care areas of intensive care and surgery (reducing the number of bladder catheterization contaminations through proper skin disinfection and wound prophylaxis); (v) the monitoring of environmental microbial contamination and the control of environmental sanitization procedures; and (vi) HAI prevention (staff and environmental hygiene).

Surveillance, prevention, and antibiotic policies are deeply interrelated and interdependent. However, the greatest effectiveness is achieved when all these measures are used in together.

## 2. Conclusions

More than half of ICAs are preventable, especially those associated with certain behaviors, through planning programs aimed to prevent and control the transmission of infections [23].

According to a recent document issued by the World Health Organization, Global Strategy on Infection Prevention and Control, “having active infection prevention and control (IPC) programmes in place is a proven effective approach to protect patients, health workers and visitors to healthcare facilities by preventing avoidable infections acquired during care provision, including those caused by antimicrobial resistant and epidemic- and pandemic-prone pathogens” [24].
